# Annual Meetings of Hospitals and Societies

**Published:** 1921-03-26

**Authors:** 


### Annual Meetings of Hospitals and Societies.

### Royal Sussex County Hospital.

Viscount Gage presided at the annual meeting of the Royal Sussex County Hospital, and congratulated the Board of Governors upon the fact that the adverse balance of £5,000 of 1919 had been converted into a credit balance of £17,053 at the end of 1920, although the surplus had to be employed for the extension fund. Patients' payments now amount to £50 a week.

### Liverpool Royal Southern Hospital.

At the annual meeting it was stated that the almoner system, started in October last, has already proved most successful. This officer assesses the in- and out-patients as to contributions, and under a regular system the revenue from this source has much improved. The institution is in debt to the extent of £10,000, and it is felt strongly that more substantial payments should be made in respect of National Health patients.

### Liverpool Royal Infirmary.

The income and expenditure account for 1920 of the Liverpool Royal Infirmary shows a balance on the wrong side of £6,907. At the annual general meeting special stress was put upon the justness of the claim that this and other hospitals have upon the National Health Insurance scheme. Patients coming from outside a certain radius have to pay £1 a week towards cost of maintenance. An appeal for funds for a new nurses' home has been postponed.

### Mount Vernon Hospital.

The Hon. Sidney C. Peel, M.P., D.S.O., presided at the annual Court of Governors of the Mount Vernon Hospital on March 16. The report states that to meet the heavy cost of maintenance the Committee have been compelled to dispose of £5,000 Exchequer Bonds. The total expenditure for the year was £24,168, and the income was £25,306. The number of in-patients admitted during the year was 360, as compared with 343 in 1919. New out-patients for the same periods totalled 2,306 and 2,330 respectively; out-patients' attendances increased from 13,970 in 1919 to 14,986 in 1920. The Committee expresses its appreciation of the gifts of clothing and material by the Ladies' Work Party, and its pleasure at the re-election of Dr. G. Basil Price, C.M.G., as an assistant physician on his return to civil life.

### The Royal Waterloo Hospital.

The Lord Mayor, who presided at the annual Court of Governors of the Royal Waterloo Hospital, held at the Mansion House on March 16, reminded those present that when founded in 1816 the hospital was located at Doctors Commons. The Lord Mayor has always been the President. Of the sum of £80,000 required to build a nurses' home and out-patient department, £21,000 has been subscribed. The general income has been well maintained, but the accumulated deficit totals £4,943, and is attributable principally to the increase of £3,188 in the expenditure. Major R. L. Bower's offer of a complete *x*-ray apparatus was gratefully accepted, and the amount of £345 received in response to an appeal for funds to purchase an *x*-ray installation will be available for hospital maintenance.

### University College Hospital.

The Duke of Bedford was re-elected as President of the University College Hospital at the annual meeting. Lord Oranmore and Browne moved the adoption of the report, which states that the total expenditure for the year amounted to £70,684, and exceeded the total income by £9,639. Annual subscriptions had increased from £3,470 to £3,732, and donations from £8,968 to £11,443. The latter amount included voluntary contributions from patients and friends, amounting to about £6,000. Under the scheme devised in connection with the gift of £835,000 from the Rockefeller Foundation to Hospital and Medical School, and of £370,000 to the College for the purpose of developing medical education in London, the Hospital and Medical School and the College are to form constituent parts of a co-operative educational centre, each with its definitely defined functions. The scheme so far as it affects the hospital involves the provision of 120 additional beds to be allotted to the medical and surgical units, and sixty additional beds for a new obstetrical unit. Of the £835,000 allotted to the Hospital and Medical School, £400,000 is to be devoted to a building programme. The additional charge on the hospital's finances will amount to approximately £20,000 per annum, and the Committee express the hope that increased financial support will be accorded by the public. The year has been an exceptionally busy one in all departments, and a record has been established both as regards the number of patients treated and the volume of work achieved.

